# Inhibitory Effects of Ketamine on Lipopolysaccharide-Induced Microglial Activation

**DOI:** 10.1155/2009/705379

**Published:** 2009-03-30

**Authors:** Yi Chang, Jie-Jen Lee, Cheng-Ying Hsieh, George Hsiao, Duen-Suey Chou, Joen-Rong Sheu

**Affiliations:** ^1^Department of Anesthesiology, Shin Kong Wu Ho-Su Memorial Hospital, School of Medicine, Fu-Jen Catholic University, 24205 Taipei, Taiwan; ^2^Department of Pharmacology, Taipei Medical University, 11031 Taipei, Taiwan; ^3^Department of Surgery, Mackay Memorial Hospital, Taipei 10449, Taiwan

## Abstract

Microglia activated in response to brain injury release neurotoxic factors including nitric oxide (NO) and proinflammatory cytokines such as tumor necrosis factor-*α* (TNF-*α*) and interleukin-1*β* (IL-1*β*). Ketamine, an anesthetic induction agent, is generally reserved for use in patients with severe hypotension or respiratory depression. In this study, we found that ketamine (100 and 250 *μ*M) concentration-dependently inhibited lipopolysaccharide (LPS)-induced NO and IL-1*β* release in primary cultured microglia. However, ketamine (100 and 250 *μ*M) did not significantly inhibit the LPS-induced TNF-*α* production in microglia, except at the higher concentration (500 *μ*M). Further study of the molecular mechanisms revealed that ketamine markedly inhibited extracellular signal-regulated kinase (ERK1/2) phosphorylation but not c-Jun N-terminal kinase or p38 mitogen-activated protein kinase stimulated by LPS in microglia. These results suggest that microglial inactivation by ketamine is at least partially due to inhibition of ERK1/2 phosphorylation.

## 1. Introduction

Microglia are a type of neuroglia that support, nurture, and protect neurons which maintain homeostasis of the fluid that bathes neurons [1]. It is an innate immune component of the central nervous system (CNS) parenchyma [2]. Under physiological conditions, residential microglia are quiescent and scattered throughout the CNS. Occasionally, microglia are moderately activated to play the classic role as scavengers for maintaining and restoring the CNS. Activated microglia release proinflammatory cytokines such as interleukin-1*β* (IL-1*β*) and tumor necrosis factor-*α* (TNF-*α*) to induce inflammatory responses [3]. Furthermore, they also release neurotoxins like reactive oxygen species (ROS) and nitric oxide (NO) [4], which amplify the inflammatory responses and cause neuronal damage in the CNS. Sustained overactivation of microglia is found in many neurodegenerative diseases such as multiple sclerosis, Alzheimer's disease, Parkinson's disease, HIV-associated dementia, and ischemia/reperfusion brain injury [5, 6].

Endotoxins are high-molecular-weight complexes of lipopolysaccharide (LPS) that are major components of the outer membranes of the cell walls of gram-negative bacteria [7]. The most severe septic microvascular inflammatory responses, however, are caused by gram-negative bacteriemia, and these responses can be produced by an injection of LPS. Endothelial injury, activation of the coagulation cascade, platelet aggregation, and thrombocytopenia have all been shown to contribute to vascular fibrin deposition in LPS-induced septic shock [7–9]. LPS also triggers a series of inflammatory reactions in microglia. LPS is known to induce NO and TNF-*α* production in microglia through various extracellular signal-regulated kinase (ERK), p38 mitogen-activated protein kinase (p38 MAPK), and c-Jun N-terminal kinase (JNK) pathways [10, 11].

Ketamine, an anesthetic induction agent, is generally reserved for use in patients with severe hypotension or respiratory depression [12]. As we described previously, ketamine (200 and 350 *μ*M) significantly inhibited platelet activation stimulated by collagen [13]. On the other hand, ketamine has been reported to exert anti-inflammatory effects on macrophages and leucocytes in in vitro and in vivo studies [14–16]. Shibakawa et al. [17] also reported that ketamine (300 ~ 1000 *μ*M) significantly inhibited some of the inflammatory responses in microglial cells stimulated by LPS. However, the detailed mechanisms underlying the anti-inflammatory effects of ketamine in microglia stimulated by LPS still have not been completely resolved yet. We therefore further examined the effect of ketamine in LPS-induced microglial activation in primary cultures from rats and utilized the findings to further characterize the anti-inflammatory effects of ketamine.

## 2. Materials and Methods

### 2.1. Materials

Ketamine, LPS (*Escherichia coli*, serotype 0127: B8), and 3-(4,5-dimethylthiazol-2-yl)-2,5-diphenyl-tetrazolium bromide (MTT) were purchased from Sigma (St. Louis, Mo, USA); RPMI-1640 medium, fetal bovine serum (FBS), trypsin (0.25%), L-glutamine, and penicillin/streptomycin were from GibcoBRL (Gaithersburg, Md, USA); deoxyribonuclease type I (DNase I) was from Roche (Indianapolis, Ind, USA); anti-ERK1/2, anti-phospho-ERK1/2 (Thr^202^/Tyr^204^), anti-JNK1/2, anti-p38 MAPK, and anti-phospho-JNK1/2 (Thr^183^/Tyr^185^) monoclonal antibodies (mAbs) were from Cell Signaling (Beverly, Mass, USA); the anti-phospho-p38 MAPK (Ser^182^) mAb was from Santa Cruz (Santa Cruz, Calif, USA); the horseradish peroxidase-conjugated secondary antibody was from Amersham (Buckinghamshire, UK); and the IL-1*β* and TNF-*α* enzyme immunoassay (EIA) kits were from R&D Systems (Minneapolis, Minn, USA).

### 2.2. Cell Cultivation

Wistar rats (7 days old; from the Experimental Animal Center, College of Medic ine, National Taiwan University) were used in this study. All animal experiments and care were performed according to the *Guide for the Care and Use of Laboratory Animals* (National Academy Press, Washington, DC, 1996). Wistar rats were deeply anesthetized with ether and transcardially perfused with normal saline until the lungs and liver were clear of blood. After perfusion, the brain was removed and kept in RPMI-1640 medium. After dissecting the meninges, the brain tissue was minced in ice-cold RPMI-1640 and treated with trypsin (0.25%) and deoxyribonuclease (10 mg mL^−1^) in RPMI-1640 for 2 hours at 37°C. Treated tissues were further minced in 10% FBS and centrifuged at 1000 rpm for 10 minutes. The tissue pellet was resuspended in RPMI-1640 and then seeded in 75 cm^2^ flasks at 37°C (95% O_2_ and 5% CO_2_).

Microglia were harvested from flasks of mixed glial cultures after shaking for 2 hours. Cells were collected by centrifugation and then seeded at 5 × 10^5^ cells mL^−1^. After incubation for 1 hour at 37°C, nonadherent or weakly adherent cells were removed by gentle shaking and washed out. Cells were further cultured in RPMI-1640 supplemented with 10% FBS for 1 day. Approximately 2 × 10^6^ cells were obtained per brain used [18]. To determine the purity of the microglia, an immunocytochemical analysis was carried out using a microglial-specific OX-42 antibody. These primary cultured cells were >95% OX-42-positive indicating that they were composed of microglia.

### 2.3. Cell Viability

Microglial viability after 24 hours of continuous exposure to different concentrations of LPS (1 ~ 200 ng mL^−1^) and ketamine (100 ~ 500 *μ*M) was measured with a colorimetric assay based on the ability of mitochondria in viable cells to reduce the MTT as previously described [19]. The percentage of cell viability was calculated as the absorbance of treated cells/control cells × 100%.

### 2.4. Determination of Nitrite Concentration

To determine NO production from microglia, nitrite (a stable oxidative end product of NO) accumulation in the media of microglia was measured using a colorimetric method [18], with minor modifications. Briefly, 100 *μ*L of supernatant was incubated with an equal volume of Griess reagent (1% sulfanilamide and 0.1% naphthyl-ethylenediamine dihydrocholoride in 2.5% phosphoric acid). After a 30-minute incubation at room temperature, the optical absorbance at 550 nm was measured with a microplate reader. Nitrite concentrations were calculated by regression with standard solutions of sodium nitrite prepared in the same culture medium [18].

### 2.5. Enzyme-Linked Immunosorbent Assay (ELISA) of IL-1*β* and TNF-*α*

For determination of IL-1*β* and TNF-*α* production from microglia, microglia (5 × 10^5^ cells mL^−1^) were plated onto 24-well culture plates for 24 hours. Cells were pretreated with various concentrations of ketamine (100 ~ 500 *μ*M) or an isovolumetric PBS buffer for 30 minutes and then treated with LPS (100 ng mL^−1^) for the indicated times (1 ~ 24 hours). After incubation with LPS, supernatants were collected and immediately frozen at −70°C. The IL-1*β* and TNF-*α* levels of the supernatants were measured using ELISA kits according to the manufacturer's protocol.

### 2.6. Western Blot Analysis

For determination of the expression of MAPKs in microglia, Western blot analyses were performed as described previously [18]. Microglia (5 × 10^5^ cells mL^−1^) were cultured on 24-well plates and treated with ketamine (100 and 250 *μ*M) or an isovolumetric PBS buffer for 30 minutes followed by the addition of LPS (100 ng mL^−1^). At indicated times, cells were washed with ice-cold PBS buffer (pH 7.3). Proteins were extracted with lysis buffer for 30 minutes. In addition, phosphatase inhibitors (10 mM sodium fluoride, 1 mM sodium orthovanadate, and 10 mM sodium pyrophosphate) were added to the lysis buffer for the phosphorylated MAPK analysis. Lysates were centrifuged, and the supernatant (50 *μ*g protein) was subjected to SDS-PAGE and electrophoretically transferred onto PVDF membranes (0.45 mm; Hybond-P; Amersham). After incubation in blocking buffer (50 mM Tris-HCl, 100 mM NaCl, 0.1% Tween 20, and 5% dry skim milk; pH 7.5) overnight at 48 hours and being washed three times with PBS buffer, blots were treated with either anti-phospho-ERK1/2 (p42/44), anti-ERK1/2, anti-phospho-JNK1/2 (p46/54), anti-JNK1/2, anti-p38 MAPK, or anti-phospho-p38 MAPK mAbs (1:2000) in a PBS buffer for 3 hours. They were subsequently washed three times with PBS buffer and incubated with a peroxidase-conjugated goat anti-mouse or anti-rabbit antibody (1:3000) for 2 hours. Blots were then washed three times, and the band with peroxidase activity was detected using film exposure with enhanced chemiluminescence detection reagents (ECL^+^ system; Amersham). Densitometric analysis of specific bands was performed using a Photo-Print Digital Imaging System (IP-008-SD) with analytic software (Bio-1Dlight, Vers. 2000).

### 2.7. Statistical Analysis

The experimental results are expressed as the means ± S.E.M. and are accompanied by the number of observations. Data were assessed by the method of analysis of variance (ANOVA). If a significant difference among the group means was noted, the difference between two groups would be assessed using the Newman-Keuls method. A *P* value of <.05 was considered statistically significant.

## 3. Results

**Figure 1:**
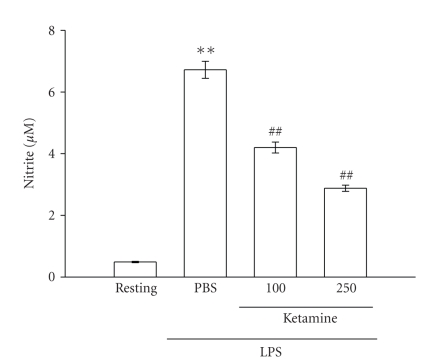
Effect of ketamine on nitrite formation in LPS-activated microglia. Microglia (5 × 10^5^ cells mL^−1^) were treated with ketamine (100 and 250 *μ*M) or an isovolumetric PBS buffer for 30 minutes, followed by the addition of LPS (100 ng mL^−1^) for 24 hours. Cell-free supernatants were assayed for nitrite production as described in Section 2. Data are presented as the means ± S.E.M. (*n* = 3). **P < .01, compared to the resting group; ^*##*^ *P* < .01, compared to the PBS-treated group.

**Figure 2:**
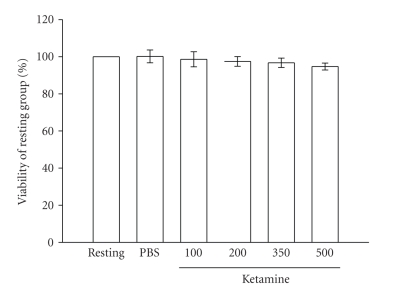
Tetrazolium dye 3-(4, 5-dimethylthiazol-2-yl)-2,5-diphenyl tetrazolium bromide (MTT) assay of ketamine-treated microglia. Microglia (5 × 10^5^ cells mL^−1^) were treated with various concentrations of ketamine (100 ~ 500 *μ*M) or an isovolumetric PBS buffer for 24 hours. Cell viability was measured by a colorimetric assay at 550 nm based on the ability of mitochondria to reduce the MTT in viable cells. Data are presented as the means ± S.E.M. (*n* = 3).

According to a preliminary test, activation of microglia by LPS (100 ng mL^−1^) induced a significant and marked increase in nitrite formation. Therefore, an LPS concentration of 100 ng mL^−1^ was employed in the following experiments. In this study, the concentration of nitrite produced in the cell supernatant time-dependently increased from 0.5 ± 0.0 (resting) to 6.7 ± 0.3 *μ*M at 24 hours after LPS treatment (Figure 1). Ketamine (100 and 250 *μ*M) concentration-dependently inhibited LPS-(100 ng mL^−1^) stimulated nitrite production by approximately 40% and 60%, respectively, (Figure 1). Ketamine neither interfered with the Griess reaction nor reacted with native NO (data not shown). These results demonstrate that ketamine markedly suppressed NO production stimulated by LPS in microglia. Furthermore, neither ketamine (100 ~ 500 *μ*M) (Figure 2) nor LPS (100 ng mL^−1^) (data not shown) significantly affected the cell viability of microglia for 24 hours according to the MTT assay.

**Figure 3:**
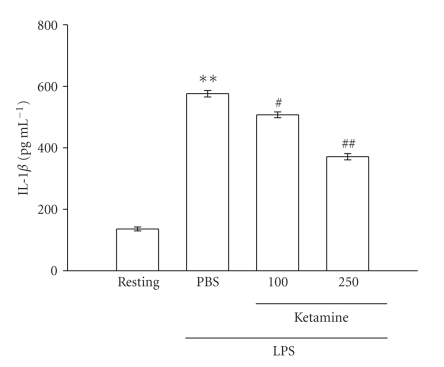
Effect of ketamine on interleukin (IL)-1*β* production in lipopolysaccharide (LPS)-activated microglia. Microglia (5 × 10^5^ cells mL^−1^) were treated with ketamine (100 and 250 *μ*M) or an isovolumetric PBS buffer for 30 minutes, followed by the addition of LPS (100 ng mL^−1^) for 12 hours. Cell-free supernatants were assayed for IL-1*β* production as described in Section 2. Data are presented as the means ± S.E.M. (*n* = 3). **P < .01, compared to the resting group; ^*#*^ *P* < .05 and ^*##*^ *P* < .01, compared to the PBS-treated group.

LPS (100 ng mL^−1^) induced a time-dependent increase in IL-1*β* formation in microglia, and it reached a maximal level at 12 hours rising from 135.9 ± 6.5 (resting) to 575.9 ± 10.6 pg mL^−1^ (Figure 3). On the other hand, after pretreatment of cells with various concentrations of ketamine for 30 minutes followed by the addition of LPS (100 ng mL^−1^) for 12 hours, we found that ketamine (100 and 250 *μ*M) concentration-dependently inhibited IL-1*β* production by approximately 13% and 36%, respectively, (Figure 3).

**Figure 4:**
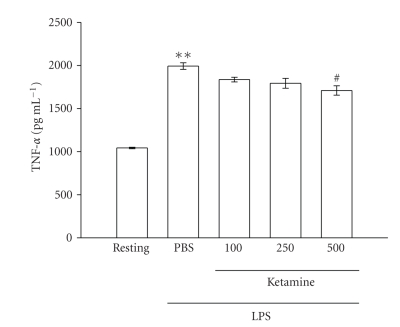
Effect of ketamine on tumor necrosis factor (TNF)-*α* production in lipopolysaccharide (LPS)-activated microglia. Microglia (5 × 10^5^ cells mL^−1^) were treated with various concentrations of ketamine (100, 250, and 500 *μ*M) or an isovolumetric PBS buffer for 30 minutes, followed by the addition of LPS (100 ng mL^−1^) for 3 hours. Cell-free supernatants were assayed for TNF-*α* production as described in Section 2. Data are presented as the means ± S.E.M. (*n* = 3). **P < .01, compared to the resting group; ^*#*^ *P* < .05, compared to the PBS-treated group.

On the other hand, activation of microglia by LPS (100 ng mL^−1^) induced a significant increase in TNF-*α* formation. The peak activation of TNF-*α* formation occurred 3 hours after LPS (100 ng mL^−1^) stimulation (resting, 1042.2 ± 8.8 pg mL^−1^; 3 hours, 1992.8 ± 38.2 pg mL^−1^, *n* = 3) (Figure 4). After pretreating cells with various concentrations of ketamine for 30 minutes followed by the addition of LPS (100 ng mL^−1^) for 3 hours, only ketamine at the higher concentration (500 *μ*M) significantly inhibited TNF-*α* production by approximately 11% (Figure 4).

**Figure 5:**
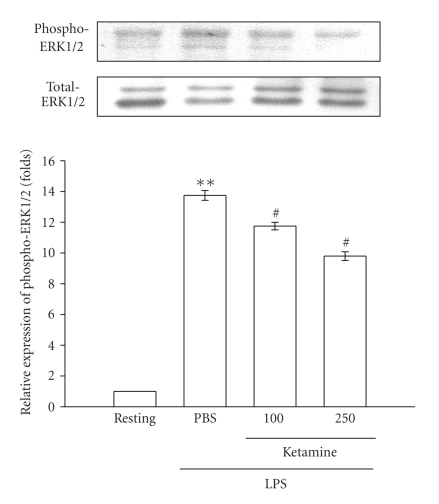
Effect of ketamine on ERK1/2 phosphorylation in lipopolysaccharide (LPS)-activated microglia. Microglia (5 × 10^5^ cells mL^−1^) were treated with ketamine (100 and 250 *μ*M) or an isovolumetric PBS buffer for 30 minutes, followed by the addition of LPS (100 ng mL^−1^) for 45 minutes. ERK1/2 phosphorylation was determined by Western blotting with a monoclonal antibody which recognizes only phosphorylated ERK1/2 (p42/p44). Data are presented as the means ± S.E.M. (*n* = 3). **P < .01, compared to the resting group; ^*#*^ *P* < .05, compared to the PBS-treated group.

**Figure 6:**
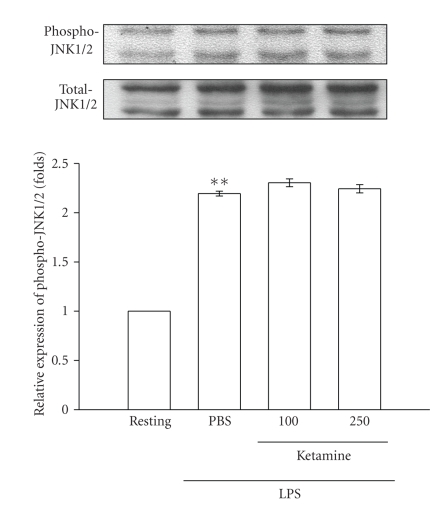
Effect of ketamine on JNK1/2 phosphorylation in lipopolysaccharide (LPS)-activated microglia. Microglia (5 × 10^5^ cells mL^−1^) were treated with ketamine (100 and 250 *μ*M) or an isovolumetric PBS buffer for 30 minutes, followed by the addition of LPS (100 ng mL^−1^) for 45 minutes. JNK1/2 phosphorylation was determined by Western blotting with a monoclonal antibody which recognizes only phosphorylated JNK1/2 (p46/p54). Data are presented as the means ± S.E.M. (*n* = 3). **P < .01, compared to the resting group.

**Figure 7:**
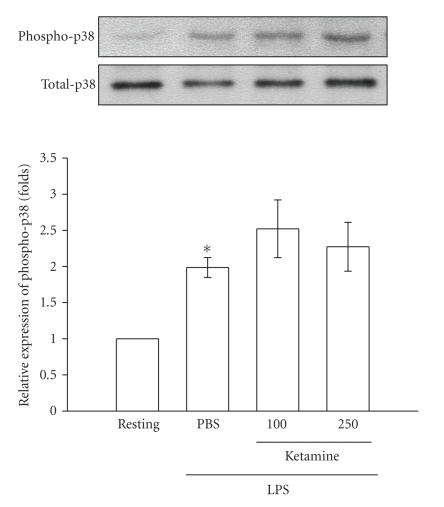
Effect of ketamine on p38 MAPK phosphorylation in lipopolysaccharide (LPS)-activated microglia. Microglia (5 × 10^5^ cells mL^−1^) were treated with ketamine (100 and 250 *μ*M) or an isovolumetric PBS buffer for 30 minutes, followed by the addition of LPS (100 ng mL^−1^) for 45 minutes. p38 MAPK phosphorylation was determined by Western blotting with a monoclonal antibody which recognizes only phosphorylated p38 MAPK. Data are presented as the means ± S.E.M. (*n* = 3). *P < .05, compared to the resting group.

To further investigate the inhibitory mechanisms of ketamine in LPS-induced microglial activation, three major MAPK signaling molecules were detected: ERK1/2, JNK1/2, and p38 MAPK. The immunoblot analysis revealed that treatment with LPS (100 ng mL^−1^) induced rapid and marked time-dependent phosphorylation of ERK1/2 (p42/p44), JNK1/2 (p46/p54), and p38 MAPK, which reached maximal levels at approximately 45 minutes and then returned to the basal level (data not shown). After being pretreated with ketamine (100 and 250 *μ*M) for 30 minutes, phosphorylated ERK1/2 stimulated by LPS (100 ng mL^−1^) was markedly inhibited by ketamine in a concentration-dependent manner (Figure 5). However, neither JNK1/2 (Figure 6) nor p38 MAPK (Figure 7) phosphorylation stimulated by LPS was significantly inhibited by ketamine (100 and 250 *μ*M).

## 4. Discussion

In this study, we demonstrate an anti-inflammatory effect of ketamine in primary cultured microglia. Ketamine exhibited more potent activity at inhibiting both NO and IL-1*β* formation than TNF-*α* in LPS-stimulated microglia. This discrepancy, however, needs to be further investigated. The high concentration (500 *μ*M) of ketamine caused no significant changes in the number of viable cells estimated by the MTT reduction assay (Figure 2). This finding excluded the possibility that the release of the inflammatory mediators was inhibited by the cytotoxic actions of ketamine.

It is known that microglia play a key role in mediating inflammatory processes in the CNS, which are associated with various neurodegenerative diseases. LPS, a glycolipid derived from the membrane surface of gram-negative bacteria (i.e., an endotoxin), can trigger a series of inflammatory reactions in phagocytes such as microglia. With LPS stimulation, microglia are activated to drastically change their cellular functions, producing various types of inflammatory mediators such as NO, TNF-*α*, and IL-1*β*. NO and TNF-*α* are two major inflammatory mediators. NO is beneficial as a messenger or modulator, but in conditions such as oxidative stress, it is potentially toxic. NO generation by activated microglia has been shown to cause excitotoxicity through inducing glutamate release and inhibiting neuronal respiration [20]. TNF-*α* and IL-1*β* released from activated microglia also stimulate NO production in glial cells and may have a direct effect on neurons through activating receptors that contain the death domains involved in apoptosis [21].

In general, total plasma levels of ketamine are reported to be in the range of 33 ~ 94 *μ*M immediately after 2.0 ~ 2.2 mg kg^−1^ i.v. administration in humans [22]. Therefore, we considered 100 *μ*M to be a higher clinically relevant concentration achievable during induction of ketamine anesthesia, assuming that protein binding is comparable in serum-supplemented media and plasma. Our studies suggest that ketamine may modulate some of the inflammatory responses (i.e., NO and IL-1*β*) of microglia stimulated by LPS in vitro at the high range of clinically achievable concentrations.

This anti-inflammatory property of ketamine may have advantages in clinical therapy. An adequate inflammatory reaction results from the equilibrium between proinflammatory and anti-inflammatory influences. Ketamine administrated in a stress situation favors this equilibrium. This is based on clinical data published several years ago. The survival rate in intensive care unit of patients with septic shock was improved when they received ketamine as a sedative [23].

MAPKs play important roles in mediating cytokine (i.e., TNF-*α* and IL-1*β*) release with LPS-stimulated microglial activation [10, 11, 24]. We thus further investigated the roles of MAPKs involved in ketamine-mediated suppression of inflammatory responses in LPS-stimulated microglia. MAPKs are a family of serine-threonine kinases activated by many stimuli including growth factors and hormones in proliferative cells [25]. MAPKs are able to regulate a number of transcription factors, cytoplasmic proteins, and downstream kinases. This family consists of three major subgroups. ERK1/2 are involved in proliferation, adhesion, and cell progression [25]. p38 MAPK and JNK1/2 or stress-activated protein kinases, which include the 46-kDa JNK1 and 54-kDa JNK2 isoforms, are involved in death signaling processes [25]. In the present study, we found that ketamine inhibited ERK1/2 but not JNK1/2 or p38 MAPK phosphorylation in LPS-stimulated microglia. Zhang et al. [26] have reported that ketamine could abolish hyperglycemia-activated ERK1/2 phosphorylation through inhibition of the N-methyl D-aspartate-mediated calcium influx, which subsequently reduces the hyperglycemia-exaggerated damage [26].

In conclusion, although ketamine has been shown to exert anti-inflammatory effects on a variety of immune cells, the exact mechanisms responsible for these actions are not well understood. Our results suggest that ketamine's anti-inflammatory activity may be mediated, at least in part, by inhibition of ERK1/2 phosphorylation in primary cultured microglia. However, more detailed anti-inflammatory mechanisms of ketamine in microglia need to be further investigated and classified.
